# On Small Pox

**Published:** 1856-10

**Authors:** N. F. Powers


### ARTICLE IV.

*On Small Pox.* By N. F. Powers, M. D.

It is highly important for the advancement of medical science that many facts of the present should be gathered together, so as to serve as data from which generalizations may be made, and inferences drawn, so as to form a basis for scientific remedial treatment.

Having attended, since March, 1849, several endemics of Small Pox in the State of Georgia, I have concluded to write some of my notions in reference to this loathsome disease. Not that I have been a discoverer, or would put upon wing some new fledged notion, or give birth to a wild theory to be only the "vision of an hour," but only that I may render up a gift on the altar of my profession, and awaken the minds of practitioners to the subject under consideration.

Small Pox, or Varioloid, is an acute inflammation of the skin and mucous membranes. Cullen classifies it among the Pyrexiœ, and order exanthemata. Many contend that it was known to the ancients; that Job and the Egyptians suffered much from boils in the early ages of the world, and that even anterior to Job's day, (though no one knows when that was,) those very ancient people, the Chinese, had the Small Pox. But others say that no intelligible account is given of it before the sixth century, and that Razes, an Arabian, about the close of the ninth century, was the first professional writer who noticed this disease.

It was universally confounded with Rubeola, until the great Sydenham pointed out their diagnostic symptoms, and divided this disease into the discreet and confluent—which terms are very significant of the intensity of the disease, but there is no specific difference in the origin of the two. The one is denominated confluent, because the pustules run together; the other, discreet, because they do not coalesce.

The history of this disease may be given under four heads, or different stages—1st. The stage of incubation. 2d. That of the eruptive fever. 3d. That of maturation. 4th. That of decline and desquamation.

The period of incubation—This is the time which classes between the exposure to the virus of Small Pox, and the time of the supervention of the eruptive fever. It may be called the latent period of the disease, as the virus remains hidden in the system, between seven and twenty-one days. In some two hundred noted cases, the period of incubation was fourteen days. This time may be lessened or prolonged by contingent circumstances. The patient enjoys, during this time, an accustomed degree of health. Why the virus remains dominant, inactive, for such a length of time, has never been satisfactorily accounted for by any pathologist.

The eruptive stage—It sets in with a rigor or chill, followed by fever, which continues for some seventy-two hours, The chill comes on suddenly, unexpectedly. In the majority of instances there are no premonitions of disease. During the fever there are pains in the *loins*, bones, in the joints and muscles of the lower extremities, so much so that I have known patients to be treated in the incipiency of this disease, for inflammatory rheumatism. There is also pain in the head, which is of the frontal variety. These pains, as in the loins, head, bones, muscles and a furred tongue, whitish in appearance, are prodromous of the disease. Chomel thinks that the lumbago is pathognomonic, and refers it to the kidneys. Indeed the whole glandular system is involved. The kidney secretes urine which is diminished in quantity, but increased in specific gravity—its color is deep red, often albuminous and turbid. The parotid and submaxillary glands are somewhat enlarged, and secrete an unusual quantity of fluid. Large quantities of bile are often vomited from the stomach—the nausea is great, and the emesis persistent—the pulse is full and frequent, varying from 100 to 120 per minute. The heat of the skin is greatly increased, though the temperature is not so great as in scarlatina. The tongue, at the onset, is whitish, and then, as the fever advances, becomes red at the point and edges—a dark fur covers it, and the papilla elevated.

In children, during the eruptive fever, convulsions supervene, which assimilate in many respects the symptoms of epilepsy.

In the course of three days of the eruptive fever, pustules make their appearance on the forehead ; then on the lips. The eruption continues to come out in successive crops until the whole surface is covered in some thirty-six hours. The papules measure from a half to two-thirds of a line in diameter, and resemble flea-bites ; they disappear under pressure. The papules having made their appearance, the nausea and vomiting cease, the fever declines, the pulse becomes natural, full and regular.

The papules about the second day change in appearance--they are more elevated ; their apices contain a small particle of serous fluid, which can be seen soon after the papule is visible by the aid of a magnifying glass. This transparent elevation corresponds to the point of umbilication in the pustule. This point in the papule enables the physician to diagnose the disease, so soon as the eruption makes its appearance. About the second day the papules change into vesicles. The fluid in the vesicles is changed in its severity and becomes opeline, purulent, or in other words, the vesicle is changed into a pustule. When a vesicle is changed to a pustule, which is about the fifth day of the eruption, a distinct areole is formed around its base ; that is, if the pustule is of a healthy character. The pustules in patients who are anermic from any cause, whether it be chlorosis, chronic, or cachectic affections, the pustules are not well filled ; they are pale and flabby ; scarcely any areole visible around their base. Such patients generally die, unless closely attended to, and stimulate when necessary.

Maturation—The pustules, before bursting, become flattened, and are about the size of a half-English pea—their contents are yellowish and purulent. The pustules can be seen in the mouth, nose, on the tongue and in the throat. The mucous membrane is red and thickened, and the tonsils swollen. I have never seen pustules on the mucous membranes of the stomach and intestines, though Rostan asserts that he has found them throughout the intestinal canal. The majority of writers disagree with him. Changes do take place, but these may be embraced under that of "Hayer's Plagues."

Pustules are often seen in the corner of the eye, and produce blindness. The pustules are more numerous on the face, hands, feet and buttocks. In children they are also numerous on the inner side of the thighs, from the irritating effects of urine. About this stage the scrotum of the adult being formed with pustules, becomes vesicular, and very much distended.

About this time secondary fever comes on, that is, if the patient be affected with the discreet variety. Sometimes it does not occur, or is not a prominent symptom. In the confluent, instead of appearing on the seventh or eighth day of eruption, it manifests itself about the tenth or eleventh day. It is then often of a lower grade, or typhoid order.

Suppuration is first seen on the face, then on the breast, and lastly, on the feet. The pus often diffuses itself in confluent Small Pox under the skin of the face, so that superficially it is a mass of purulent matter. Previous to this formation, the face and whole body itches; feel hot; the skin is stiff and thick; the eyelids are greatly swollen, and cannot be opened, and hence a lonely feeling comes over the patient, which is most intolerable. The nostrils are shut, full and distended, consequently the patient has to breathe through the gaping mouth, and over the thickly pustulated tongue, which is dry and swollen. The voice is then hoarse; the pharynx and fauces being pustulated. According to Sydenham, if ten thousand pustules be on the entire body, two thousand will be on the face. This numerous pustulation is the cause of the suppuration being so extensive.

The *period* of decline and desquamation—Desiccation commences about the eighth day of eruption, and is manifested by the commencement of the drying up of the pustules, so as to form scales or scabs. The pustules burst, and in drying, their pus forms a dark brown scab. About this period there is an odor which arises from the patient, which is peculiar, fetid, mummy like, and is decidedly characteristic of the disease.

The tumefaction subsides; the appetite returns; the patient eats, from soreness and the distention of mouth, with difficulty. The scabs begin to fall off about the fourteenth day of eruption, and continue until about the twenty-fifth or fortieth day. After the period of desquamation closes, the whole surface of the body, in confluent Small Pox, is covered with a new, tender scarf skin, and sometimes the patient then has to suffer from crops of boils, situated on various parts of the body.

During the period of desquamation in confluent Small Pox, all the hair, sometimes the toe and finger nails, fall off—leaving the patient sans hair, sans nails, sans skin, sans nearly everything but appetite.

Morbid Anatomy—The pustules are found on the entire integument, on the mucous membranes about the outlets of the body. The seat of the pustules greatly modifies their umbilications. This cannot be so plainly seen on the face, hands and feet, but nevertheless, the umbilication exists. For if the hand or foot be placed in hot water about the fourth day of the eruption the pustule can be removed entire; when it will resemble a dried disc of mixed pus and blood, with a depression in the center. This depression is the diagnostic symptom of the Small Pox pustule. I have never seen the least difficulty diagnosing the disease, after the eruption had made its appearance, by the use of a magnifying glass. Though I once knew several physicians who made an error in diagnosis, from the fact, that the patient was suffering simultaneously, from Small Pox and Purpura Hœmorrhagica. Each pustule then was filled with blood, but nevertheless, the contents of the original pustule was there and could be seen. In this instance I was guided by scent more than sight.

A well formed pustule is divided into two chambers, a superficial and a deep seated one. The epidermis covers the apices of the pustule, the derm is its base; the false membrane its septum. The two chambers communicate with each other. The surface at the base of the pustule is red and highly vascular. The cause of the umbilication of the pustule, is the attachment of the false membrane to the epiderme.

Diagnosis—It is difficult during the incipiency of Small Pox, to diagnose it. There is great variety of fevers which resembles it during their onset. Pains in the loins, limbs, head, bones, joints, staggering as in drunkenness, white furred tongue are more common in varioloid than in any other disease. Its eruption resembles some little that of Rubeola, though its papules are more elevated and rougher. After the eruption makes its appearance the difficulty ceases. The transparency on the top of the vesicle—the umbilication of the pustules are very plain manifestations of Small Pox, and can be seen as other disease.

Prognosis is generallyfavorable unless the patient is *anæmic;* in the last *stages* of *pregnancy*, or *very old.*

Irregular forms of the disease are generally fatal, as when complication with diffused hemorrhage, &c.

When the patient is about to die with the disease, the breathing becomes stertorous, the pustules are flattened—white around the base—the blood looks dark under the finger and toe nails; and the shoulders are elevated at each inspiration.

Cause. This is universally acknowledged to be contagion, capable of being propagated by epidemic influences. Some say it is a specific animal poison which can be taken from the dead subject, (as in my own case,) or from the cloths, bed, bedding, from the walls of the sick-room even after the elapse of years.

In testimony of this fact, I will take the privilege of publishing a private letter from one of the oldest and most reliable practitioners of the State. I do not give his name as I am not authorised, but; nevertheless, I feel very thankful to him for his valuable information. He writes me as follows:

"You wish the facts in relation to the recent cases of Small Pox in Mr. Hawkins' family. I was not the attending physician, but Dr. B. V. Willingham who had been attending Mrs. Hawkins for sometime on account of a bad state of general health, when the eruption came out, he (Dr. Willingham) pronounced it Small Pox. I was then called in and concurred in opinion with him. It was a case of Distinct Small Pox, in a mild form, and went through the regular stages. Mrs. Hawkins had never been vaccinated—a negro woman who had been vaccinated, and who waited on Mrs. Hawkins, and slept in the room with her every night, had Varioloid. These were all the cases occurring in the family, so far as I know. Several persons had been to see Mrs. H. before the eruption came out, and some after it made its appearance, but none took it. Mrs. H. must have taken it from the house, or something (I know not what,) about it, as there was no opportunity of getting it otherwise. Mr. H.'s house, or the bed-room, is a log body, weatherboarded and ceiled. In this room the white family, who had Small Pox in 1851, were confined."

