# Errata

**Published:** 1856-12

**Authors:** 


### ERRATA.

October Number, Article on Small Pox, by N. F. Powers, M. D., on page 77, 7th line, read "elapses" instead of "classes;" page 77, 9th line, 3d paragraph, there should be a parenthesis (as in the loins, head, bones, and muscles); page 77, last line 3d paragraph, "a dark fur covers it and its papillæ;" page 78, 9th line, 3d paragraph, "The fluid is changed in its serosity;" page 78, 14th line, 3d paragraph, "anæmic" instead of "anermic;" page 78, last line 3d paragraph, "and are stimulated" when necessary; page 78, last line on the page, "Hager's Plagues;" page 79, 1st page, "seen on the cornea of the eye;" page 80, 11th line 2d paragraph, "difficulty in diagnosing;" page 80, 2d line 4th paragraph, "There is a great variety;" page 81, 2d line, "can not be seen in other diseases;" page 81, 4th line, "in the last stages of pregnancy, or in the very old;" page 81, 5th line, "as when complicated with diffused hemorrhage;" page 81, 14th line of the letter, "soon after it made its," &c.

[ARTICLE TO BE CONTINUED.]

